# Modes of Brain Cell Death Following Intracerebral Hemorrhage

**DOI:** 10.3389/fncel.2022.799753

**Published:** 2022-02-03

**Authors:** Yan Zhang, Suliman Khan, Yang Liu, Ruiyi Zhang, Hongmin Li, Guofeng Wu, Zhouping Tang, Mengzhou Xue, V. Wee Yong

**Affiliations:** ^1^Department of Cerebrovascular Diseases, The Second Affiliated Hospital of Zhengzhou University, Zhengzhou, China; ^2^Henan Medical Key Laboratory of Translational Cerebrovascular Diseases, Zhengzhou, China; ^3^Department of Emergency, Affiliated Hospital of Guizhou Medical University, Guiyang, China; ^4^Department of Neurology, Affiliated Tongji Hospital of Tongji Medical College, Huazhong University of Science and Technology, Wuhan, China; ^5^Hotchkiss Brain Institute and Department of Clinical Neurosciences, University of Calgary, Calgary, AB, Canada

**Keywords:** intracerebral hemorrhage, cell death, apoptosis, necrosis, ferroptosis, autophagy, parthanatos

## Abstract

Intracerebral hemorrhage (ICH) is a devastating form of stroke with high rates of mortality and morbidity. It induces cell death that is responsible for neurological deficits postinjury. There are no therapies that effectively mitigate cell death to treat ICH. This review aims to summarize our knowledge of ICH-induced cell death with a focus on apoptosis and necrosis. We also discuss the involvement of ICH in recently described modes of cell death including necroptosis, pyroptosis, ferroptosis, autophagy, and parthanatos. We summarize treatment strategies to mitigate brain injury based on particular cell death pathways after ICH.

## Introduction

Intracerebral hemorrhage (ICH) refers to non-traumatic bleeding into the parenchyma of the brain. It accounts for approximately 15% of cerebral vascular diseases. However, the number of deaths (2.8 million) globally due to hemorrhagic stroke is similar to that documented for the more common ischemic stroke (2.7 million), according to a systematic analysis in 2019 for the Global Burden of Disease Study (GBD 2016 Stroke Collaborators, 2019). The case-fatality rate for ICH ranges from 35% at 7 days to 59% at 1 year, while 40% of survivors regain functional independence (de Oliveira et al., 2016; Tschoe et al., 2020; Zhang et al., 2022).

The poor outcome in ICH is attributed to the primary brain injury caused by the hematoma and its mechanical compression of the brain (Shao et al., 2019) and to subsequent secondary brain injury. The latter comprises neuroinflammation and oxidative stress-mediated through a series of events initiated by the primary injury (Zhao and Aronowski, 2013; Xi et al., 2014). Both the primary and secondary brain injuries incur significant cell death and loss of neurological functions.

Given the physical compression of the hematoma, many studies have explored the effect of surgical removal of blood clots; however, no significant benefits have been found for patients with ICH (Xi et al., 2014). Similarly, there are limited medical treatments available to ameliorate secondary brain injury effectively (Bai et al., 2020a). Therefore, a better understanding of the mode of cell death in ICH should provide new insights to counter the pathology of ICH. It could result in more effective and targeted neuroprotective or neurorestorative therapeutic strategies (Guo et al., 2020).

## Pathophysiology of Brain Damage After ICH

**FIGURE 1:**
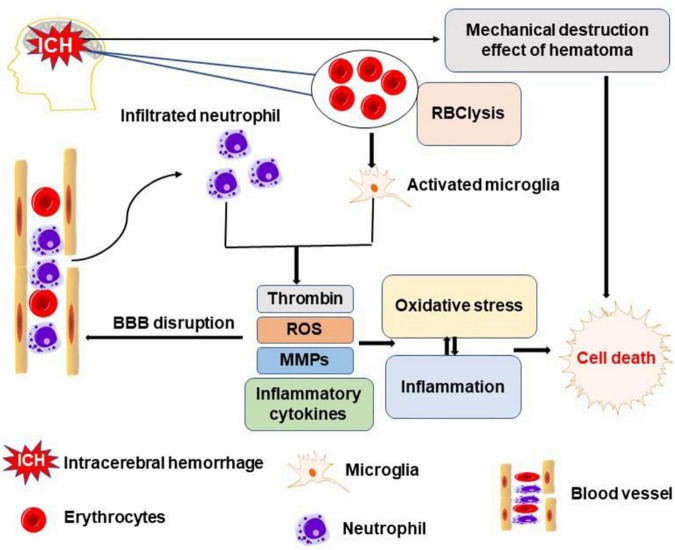
Schematic representation of major pathways leading to brain cell death after intracerebral hemorrhage (ICH). Mechanical compression of brain tissue by the hematoma directly leads to brain cell death. The degradation products of erythrocytes activate microglia which, together with invading neutrophils, release toxic substances, such as thrombin, reactive oxygen species (ROS), matrix metalloproteinases (MMPs), and inflammatory cytokines. These events of neuroinflammation and oxidative stress culminate in neuronal and glial cell death, vasogenic edema, and breakdown of the blood-brain barrier.

The extravasated blood in ICH produces primary brain injury through physical compression of local structures and altered intracranial pressure (Wilkinson et al., 2018). In addition, the hematoma and the degradation products of erythrocytes (such as hemoglobin, heme, and iron) activate microglia. The latter, together with invading neutrophils, release toxic substances, such as thrombin, reactive oxygen species, matrix metalloproteinases, and inflammatory cytokines (Figure 1), which constitute neuroinflammation and oxidative stress (Bai et al., 2020b). These collectively induce neuronal and glial cell death, vasogenic edema, and breakdown of the blood–brain barrier (Duan et al., 2016).

Besides promoting cell death, oxidative stress increases the level of proinflammatory cytokines, such as tumor necrosis factor (TNF) and interleukin-6 (IL-6), and it also upregulates inflammatory molecules, such as vascular cell adhesion molecule-1. Correspondingly, inflammatory stimuli induce the release of peroxiredoxin 2, which may act as a redox-dependent inflammatory mediator to activate macrophages to produce and release TNF (Hussain et al., 2016). The enhanced reactive oxygen species generated by polymorphonuclear neutrophils at the site of inflammation causes endothelial dysfunction that includes the opening of interendothelial junctions, which promotes the migration of inflammatory cells across the endothelial barrier (Mittal et al., 2014). In addition, signaling pathways culminating in nuclear factor-kappa B (κB) activation are influenced by reactive oxygen species to produce proinflammatory molecules on one hand and upregulation of antioxidant proteins on the other hand (Blaser et al., 2016).

Thus, ICH-related poor outcomes are due to several molecular mediators of injury that are contributed by the extravasated blood and cells reacting to the injury (Figure 1).

## Modes of Brain Cell Death Following ICH

Cell death after ICH has traditionally been ascribed to necrosis and apoptosis that occur not only at the impact site, but also distally as a result of intracranial hypertension, hypoxia, or disturbances of microcirculation. The mode of cell loss may be due to ferroptosis, necroptosis, pyroptosis, autophagy, and parthanatos according to the criteria of the Nomenclature Committee on Cell Death reported in 2009 and 2018 (Kroemer et al., 2009; Galluzzi et al., 2018). These modes of cell death exhibit distinct characteristics that can be distinguished by several experimental methods (Table 1).

**TABLE 1:** Markers to distinguish features of cell death.

Mode	Features	Markers for detection	Notes
Apoptosis	Apoptosome	Transmission electron microscope	TUNEL staining detects DNA fragment. Thus, TUNEL positive cells are not always apoptotic cells as necrotic cells may also be TUNEL-positive (Majtnerova and Rousar, 2018). Caspase activation is not a specific indicator of apoptosis, as there is also a caspase-independent form of intrinsic apoptosis; the inhibition of caspases may not prevent cell death but changes morphology (Kroemer et al., 2009). Moreover, caspases also have physiological functions for life rather than just for cell death, such as cell differentiation, DNA repair and thrombopoiesis (Galluzzi et al., 2008). Annexin V should be combined with markers of disrupted membranes such as ethidium homodimer or propidium iodide to better discriminate the different modes of cell death (Zille et al., 2019)
	DNA fragmentation	DNA ladders; FACS quantification of hypodiploid cells; TUNEL staining; *In situ* nick translation technique	
	Activation of proapoptotic Bcl-2 family proteins and caspases	FACS/IF/Immunoblotting with specific antibodies; Colorimetric/fluorogenic substrate-based assays in live cells	
	Phosphatidylserine exposure	FACS quantification of Annexin V binding	
Necrosis	Necrotic morphology changes (e.g., swelling of cells and organelles, appearance of vacuoles, and plasma membrane rupture followed by leakage of cellular contents)	Transmission electron microscope	Necrotic cell death is largely identified in negative terms by the absence of apoptotic or autophagic markers (Kroemer et al., 2009)
	Activation of calpains and cathepsins	Colorimetric/fluorogenic substrate-based assays in live cells; Colorimetric/fluorogenic substrate-based assays of cell lysates in microtiter plates	
	Plasma membrane rupture	Colorimetric/fluorogenic substrate-based assays of cell lysates in microtiter plates; Propidium iodide staining	
	ATP levels	Luminometric assessments of ATP/ADP ratio	
Necroptosis	Activation of RIP1/RIP3/pMLKL	Immunoblotting quantification with specific antibodies	RIPK1 autophosphorylation at serine 166 as detected by phospho-specific antibodies is a more appropriate measure of RIPK1 kinase activity and necroptosis than total protein level (Zille et al., 2019)
Pyroptosis	NLRP1/NLRP3 inflammasome	Immunoblotting quantification with specific antibodies; Quantitative Real-Time PCR	Pyroptosis depends on the formation of plasma membrane pores by members of the gasdermin protein family, often (but not always) as a consequence of inflammatory caspase activation (Galluzzi et al., 2018)
	Cleavage of caspase and gasdermin	FACS/IF/Immunoblotting with specific antibodies; Colorimetric/fluorogenic substrate-based assays in live cells	
	Level of IL-1β and IL-18	ELISA; Quantitative Real-Time PCR	
Autophagy	Autophagosomes/autophagolysosome	Transmission electron microscope	The increase of autophagosome does not completely mean enhanced autophagy, but may also be the inhibition of autophagy due to the blocked fusion of autophagosomes with lysosomes (Kroemer et al., 2009)
	LC3-I/LC3-II conversion; Activation of Cathepsin D	IF microscopy with GFP-LC3 fusion protein; Immunoblotting quantification with specific antibodies	
Ferroptosis	Mitochondrial fragmentation and cristae enlargement	Transmission electron microscope	The secondary products of lipid peroxidation, such as malondialdehyde and 4-hydroxynonenal, are stable markers to assess lipid peroxidation because lipids peroxides are difficult to directly detect due to their super activity and short lifetime (Zheng et al., 2021)
	Iron deposition	Perls staining	
	Lipid peroxides	ELISA; BODIPY; Liperfluo; liquid chromatography and tandem mass spectrometry analysis	
	Activation of GSH, GPX4, NADPH, SLC7A11, p53, ACSL4, LPCAT3, TfR1, DMT1, STEAP3 and ferritin	FACS/IF/Immunoblotting with specifical antibodies	
Parthanatos	Apoptosis inducing factor nuclear translocation	Transmission electron microscope; IF microscopy with specific antibodies	Apoptosis inducing factor mediated caspase-independent cell death also implicates other molecules such as PARP-1, calpains, Bax, Bcl-2, histone H2AX, and cyclophilin A (Delavallee et al., 2011)
	Activation of Poly (ADP-ribose) polymerase-1	FACS/IF/Immunoblotting with specific antibodies	
	Accumulation of poly (ADP-ribose)	Immunoblotting quantification with specific antibodies	

*FACS, fluorescence-activated cell sorter; IF, immunofluorescence; RIP, receptor-interacting protein; pMLKL, phosphorylated mixed lineage kinase domain-like pseudokinase; GSH, glutathione; GPX4, glutathione peroxidase 4; SLC7A11, solute carrier family 7 member 11; ACSL4, Acyl-CoA synthetase long-chain family member 4; LPCAT3, lysophosphatidylcholine acyltransferase 3; TfR1, transferrin receptor 1; DMT1, divalent metal (ion) transporter 1; STEAP3, six-transmembrane epithelial antigen of prostate 3.*

### Apoptosis

**FIGURE 2:**
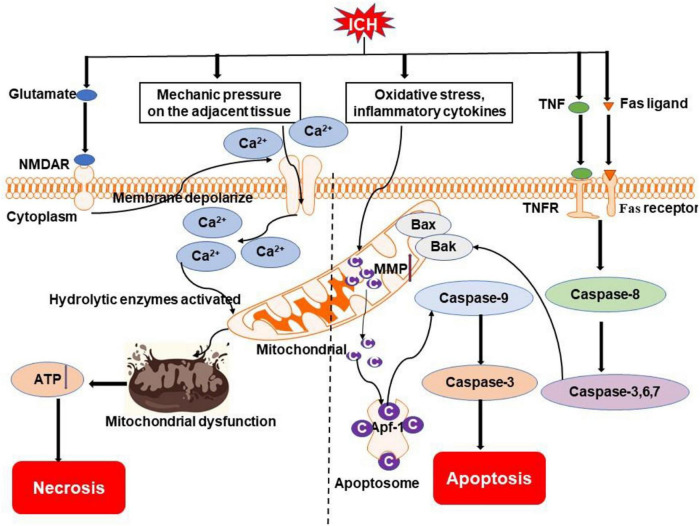
Overview of apoptosis and necrosis pathways following ICH. Oxidative stress, inflammatory cytokines (e.g., TNF), and Fas/Fas ligand after ICH may activate intrinsic caspase-dependent pathways to induce the emergence of mitochondrial membrane permeability (MMP). Cytochrome c is then released from mitochondria to activate caspases to initiate the process of cell death. Mechanical compression by the hematoma on adjacent tissue and activation of NMDAR by excessive glutamate after ICH can result in an influx of calcium, which causes mitochondrial dysfunction. Ultimately, cells go to die due to insufficient ATP produced by mitochondria. NMDAR, N-methyl-D-aspartate receptor; TNF, tumor necrosis factor; TNFR, TNF receptor; Bak, Bcl-2 homologous antagonist killer; Bax, Bcl-2 associated X protein; Ca^2+^, calcium ion; C, cytochrome c; Apaf-1, apoptotic protease-activating factor 1.

Apoptosis is initiated by perturbations of the extracellular or intracellular microenvironment, demarcated by permeabilization of the mitochondrial outer membrane, and promoted by executioner caspases (Sekerdag et al., 2018; Figure 2). Apoptosis involves a single cell and results in cell shrinkage and apoptosome formation, with subsequent phagocytosis by adjacent normal cells (Salihu et al., 2016).

Leukocytes infiltrating the brain in ICH can release harmful substances, such as proteolytic and oxidizing agents as well as cytokines, which can injure or kill cells through caspase-dependent or independent pathways (Young et al., 1989). Accumulating preclinical and clinical evidence shows that apoptosis is involved in the pathophysiological process of ICH (Chu et al., 2014; Salihu et al., 2016).

*In vitro*, exposure of primary cortical neurons to hemoglobin induced their activation of caspase-3 and -9 (Wang et al., 2002). Neuron, but not microglia, accumulation of extravasated serum proteins after intracerebral hemolysate exposure was accompanied by cytochrome c release and DNA fragmentation (Matz et al., 2000). In a rabbit ICH model, the levels of active caspase-3, Fas, FasL, and active caspase-8 were upregulated in neurons adjacent to the hematoma (Xu et al., 2016). Moreover, the release of cytochrome c from mitochondria increased at 1 and 3 days after ICH and returned toward baseline by day 7 *in vivo* (Felberg et al., 2002). A large proportion of cells trapped within the matrix of the hematoma were shrunken dark cells. A high number of terminal deoxynucleotidyl transferase-mediated dUTP nick end-labeling (TUNEL)-positive cells, which were used to identify cells with damaged DNA (Crowley et al., 2016c; Majtnerova and Rousar, 2018), were observed in the matrix of the hematoma, but not in the perihematoma regions (Qureshi et al., 2001). The peak time of apoptosis may vary in different ICH models. Our group found that TUNEL-positive cells were observed maximally at 2 days in the blood injection model, at 3 days in the vessel avulsion model, and between 1 and 7 days in the collagenase injection model; TUNEL-positive cells were evident in a small quantity at 21–28 days in all the three models (Xue and Del, 2003).

Clinical studies show the evidence of apoptosis in ICH. In surgical specimens obtained from 12 patients with ICH, the mean number of TUNEL-positive cells in the perihematoma region was 38% (range, 0–90%) (Qureshi et al., 2003). TUNEL-positive cells were observed in specimens obtained within 1, 2, and 5 days after the onset of symptoms (Qureshi et al., 2003). In another clinical study, the expression of apoptotic proteins was increased gradually after 6 h, reached a peak at 12–24 h, and decreased thereafter in surgical specimens of the perihematomal region (Guo et al., 2006). Furthermore, a large number of TUNEL-positive cells with the morphology of apoptosis were present in the center and periphery of the hematoma. Double staining suggested that these TUNEL-positive cells were mostly neurons and astrocytes (Matsushita et al., 2000).

### Necrosis

Necrosis is characterized by cellular swelling, plasma membrane rupture, and subsequent loss of intracellular contents and lysis. It involves the death of a group of cells and it evokes a significant inflammatory response (Bobinger et al., 2018). A necrotic cell can be detected by propidium iodide staining. Propidium iodide is a small fluorescent molecule that intercalates in DNA; it cannot passively traverse into cells that possess an intact plasma membrane (Crowley et al., 2016d). The level of propidium iodide fluorescence in a cell is directly proportional to the DNA content of that cell (Crowley et al., 2016a). Propidium iodide readilyenters into necrotic cells, but it is excluded from apoptotic cells. Hence, apoptotic cells can be distinguished from necrotic cells by costaining with propidium iodide (necrosis) and annexin V (apoptosis) (Crowley et al., 2016b).

Mechanisms of cell necrosis after ICH include mechanical pressure on the adjacent tissue, toxic chemicals produced by the metabolism of hematoma, free radicals, and lack of oxygen. Moreover, excitotoxic glutamate release is increased, but uptake is reduced after ICH. This excessive glutamate sustains the activation of postsynaptic neuronal N-methyl-D-aspartate (NMDA) receptors, promotes Ca^2+^ influx and intracellular Ca^2+^ overload, thereby causing mitochondrial dysfunction and the activation of lethal signaling pathways (Sharp et al., 2008; Li et al., 2018a; Zhou et al., 2020). In addition, infiltrating neutrophils and activated microglia/macrophages following ICH release proteolytic and oxidizing agents that attack cells with bound complement, a plasma protein that adheres to damaged cells (Galluzzi et al., 2018; Figure 2).

In a collagenase-induced ICH mouse model, plasmalemma permeability was first detected in the lesion between 1 and 3 h, with a peak of 48–72 h. Propidium iodide-positive cells were detected as early as 1 h and peaked between 48 and 72 h. At early time points, propidium iodide-positive cells were mainly distributed within the periphery of the hemorrhagic lesion, but at 24–48 h, a number of these cells were also found in core regions. The features of necrosis at 24 h after ICH were confirmed ultrastructurally by electron microscopy (Zhu et al., 2012). Another report demonstrated that propidium iodide-positive cells were evident in brain regions within 1 mm of the hematoma margin at 3 and 6 days and persisted 28 days post-ICH (Li et al., 2018c).

Clinical research shows necrosis in brain specimens 6 h after ICH onset, which is aggravated after 12 h (Zille et al., 2017). The mean proportion of shrunken eosinophilic neurons in the perihematoma region in each patient specimen was 25% (range, 0–100%) (Qureshi et al., 2003). The authors found an excess of TUNEL-positive cells, in comparison with shrunken eosinophilic cells, in 6 of 12 patients who underwent hematoma evacuation. Furthermore, the shrunken eosinophilic cell was the predominant finding for only one patient, who underwent late surgical evacuation on day 5. The authors concluded that brain cell apoptosis represents a prominent form of cell death associated with ICH in humans (Qureshi et al., 2003). However, this study has shortcomings including the small sample size, the exact size of the biopsies was not recorded, and only TUNEL staining was applied to evaluate apoptosis. Therefore, the results need further confirmation.

### Necroptosis

**FIGURE 3:**
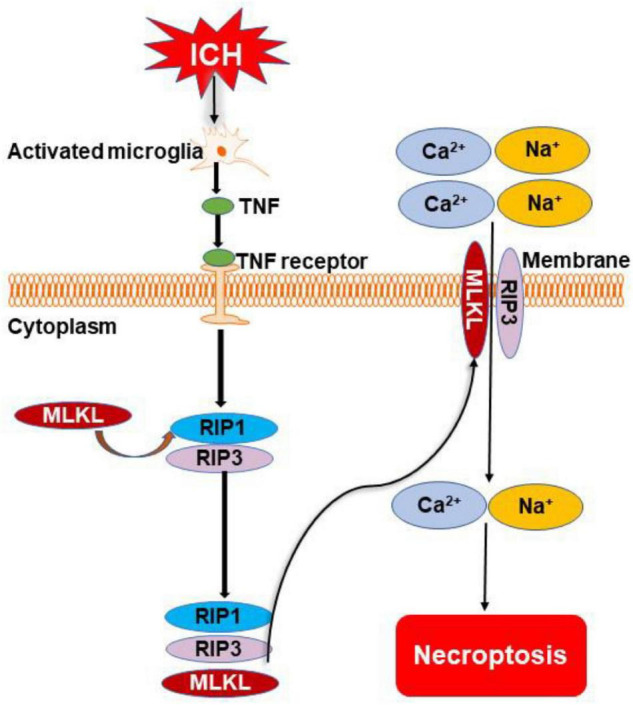
Overview of necroptosis pathways following ICH. Microglia are activated after ICH. They release tumor necrosis factor (TNF) to initiate necroptosis by binding to TNF receptor. Phosphorylation of receptor-interacting protein 1 (RIP1) to develop necrosome also occurs. The necrosome cooperates with receptor-interacting protein 3 (RIP3) for the recruitment of mixed lineage kinase domain-like protein (MLKL). The complex of MLKL and RIP3 transfers to the cell membrane and forms a channel to cause the inward flow of Ca^2+^ and Na^+^. Finally, the cell dies through the necroptotic pathway.

Necroptosis is a newly identified type of programmed necrosis that occurs through a caspase-independent mechanism initiated by the activation of receptor-interacting protein 1 (RIP1), receptor-interacting protein 3 (RIP3), and the phosphorylation of mixed lineage kinase domain-like protein (MLKL) (Lule et al., 2021; Figure 3). Morphological characteristic of necroptosis shares features of apoptosis and necrosis and is indicated by propidium iodide staining (Galluzzi et al., 2018). Evidence showed that RIP1 and RIP3 were elevated *in vitro* and *in vivo* (Su et al., 2018), indicating necroptosis is involved in the pathology process of ICH; the inhibition of necroptosis was protective in experimental ICH models (Zille et al., 2017).

Necroptosis could be induced in cultured neurons by conditioned medium from microglia stimulated with oxygen hemoglobin (Shen et al., 2017) and hemin through depleting glutathione in mouse astrocytes (Laird et al., 2008). Microglia are activated in ICH and release TNF to initiate necroptosis by binding to TNF receptor-1 and phosphorylating RIP1 to develop necrosome; this effect was inhibited by TNF inhibitor and mutation of the serine kinase phosphorylation site of RIP1 (Shen et al., 2017; Figure 3). Moreover, activated microglia also promote neuronal necroptosis through secreting exosomes and negatively regulating the expression of activating transcription factor 4 mediated by miR-383-3p in ICH rats and cells (Wei M. et al., 2021). Hemin-induced neuronal necroptosis mediated by RIP1/RIP3 (Su et al., 2018) and interleukin-1 receptor, which can form a complex with necrosome in ICH mice and primary cultured neurons (Chu et al., 2018). These results implicate activated microglia and hematoma metabolites as prominent factors leading to necroptosis. Whether thrombin, iron, and other hematoma components cause necroptosis is yet to be elucidated.

### Pyroptosis

**FIGURE 4:**
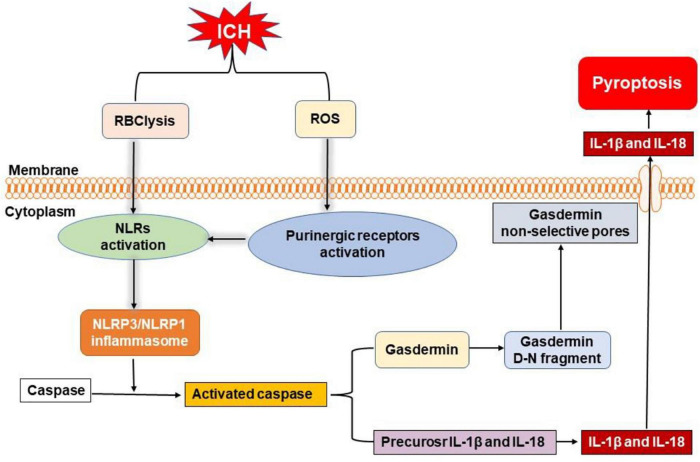
Overview of pyroptosis pathways following ICH. Nucleotide-binding oligomerization domain-like receptors (NLRs) can be activated by the degradation products of erythrocytes (such as hemoglobin, heme, and iron) and activated purinergic receptors *via* ROS, leading to the formation of inflammasome. Then, NLR pyrin domain-containing 3 (NLRP3) and NLRP1 inflammasome activate caspase to initiate the cleavage and activation of interleukin-1β (IL-1β) and interleukin-18 (IL-18). Moreover, active caspase can also cleave gasdermin to form gasdermin D-N fragment, which causes non-selective pores in membrane, inducing the release of mature IL-1β and IL-18 to elicit neuroinflammation and cell death after ICH.

Pyroptosis is gasdermin-dependent cell death, often (but not always) as a consequence of inflammatory caspase activation (Galluzzi et al., 2018). It can be triggered by nucleotide-binding oligomerization domain-like receptors (NLRs) (Galluzzi et al., 2018). NLR pyrin domain containing 3 (NLRP3) and NLRP1 inflammasome activate caspase-1 to initiate the cleavage and activation of interleukin-1β (IL-1β) and IL-18 leading to neuroinflammation and cell death after ICH (Bobinger et al., 2018; Figure 4). Liu J. et al. (2021) found that the levels of NLRP3, cleaved caspase-1, cleaved caspase-8, and IL-1β were significantly increased 3 days post-ICH in mice. NLRP3- and caspase-1-positive cells were microglia, as determined through double immunofluorescence labeling. The authors also observed bubbles and large holes in the plasma membrane of microglia from the ICH group, in contrast to the linear and intact membrane from the sham-operated group, using a transmission electron microscope (Liu J. et al., 2021). Similar results were reported by Lin et al. (2018), who demonstrated that NLRP3, caspase-1, and mature IL-1β expression were increased 24 h after ICH. NLRP3 inflammasome could be activated by endoplasmic reticulum stress-induced by ICH and it aggravated neuronal pyroptosis through increasing the expression of IL-13 (Chen et al., 2020).

Yan et al. (2021) reported that NLRP1 inflammasome expression was significantly upregulated at 6 h, with a peak at 72 h, in an autologous whole blood-induced ICH mouse model. Furthermore, these authors showed that the C-C chemokine ligand 5 (CCL5) and C-C chemokine receptor 5 (CCR5), the upstream mediators of NLRP1, were significantly increased at 3 h, peaked at 24 h, and started to decrease at 72 h after ICH. They also found that CCR5 activation promoted NLRP1-dependent neuronal pyroptosis determined by cleaved caspase-1-positive neurons, through activation of the CCR5/protein kinase A (PKA)/cyclic adenosine monophosphate (cAMP) response element-binding protein (CREB)/NLRP1 signaling pathway (Yan et al., 2021). In agreement with this study, expression of NLRP1 inflammasomes was significantly elevated at 12 h and peaked at 72 h after ICH; moreover, the expression of melanocortin receptor 4 was increased at 6 h, peaked at 24 h, and decreased at 72 h after ICH when compared to the sham group. Activation of melanocortin receptor 4, the upstream mediator of NLRP1, mitigated the ICH-induced cleaved caspase-1-positive neurons in the perihematomal region (Chen S. et al., 2019).

### Autophagy

**FIGURE 5:**
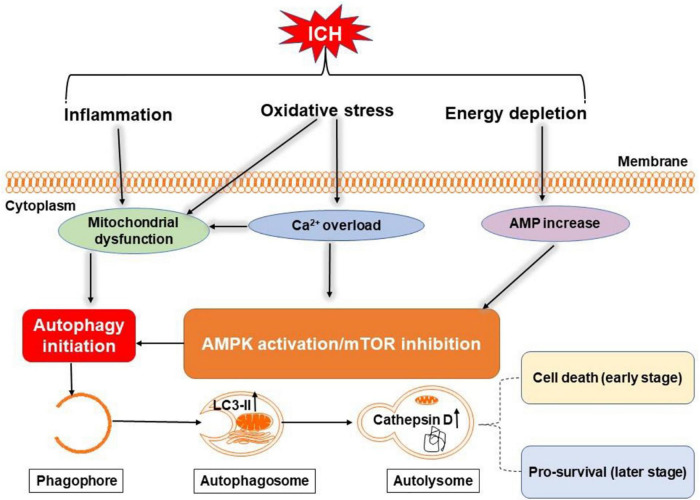
Overview of autophagy pathways following ICH. Oxidative stress, inflammation, and energy depletion after ICH may induce autophagy by activating adenosine monophosphate-activated protein kinase (AMPK) or inhibiting mammalian target of rapamycin (mTOR) and damaging mitochondria. During autophagy, light chain 3-II (LC3-II) in autophagosome and cathepsin D in lysosomes are increased. Excessive autophagy may be detrimental in the early stage of ICH, but maybe neuroprotective in the later stages through clearance of cellular debris.

Autophagy is an intracellular lysosome-mediated catabolic mechanism that is responsible for the bulk of degeneration and recycling of damaged or dysfunctional cytoplasmic components and intracellular organelles (Yuan et al., 2017). Oxidative stress, inflammation, and accumulation of free iron after ICH may induce autophagy (Bobinger et al., 2018; Figure 5). At the same time, autophagy can promote microglia activation through beclin-1-Atg5 and nuclear factor-κB pathway to exacerbate inflammation response in ICH (Yuan et al., 2017; Li et al., 2018b).

In recent years, a proliferation of experimental studies has demonstrated that autophagy plays a crucial role in cell death after ICH (Yu et al., 2017; Wu et al., 2019). One study reported that at 6 h after ICH, neuronal autophagy was prominent, but returned to baseline at 7 days after ICH (Duan et al., 2017). In another study, the ratio of light chain 3 (LC3)-II to LC3-I in the ipsilateral basal ganglia was elevated by day 3 after thrombin infusion, indicative of autophagy. Levels of cathepsin D, another sign of autophagy in lysosomes, increased at 3 days and decreased by 7 days after thrombin infusion, which follows a similar time course to the LC3-II to LC3-I conversion ratio (Hu et al., 2011).

The exact role of autophagy in ICH remains to be thoroughly understood (Li et al., 2018b). In a mouse ICH model, autophagy was reported to drive prominent brain injury (Shen et al., 2016). Another study found that ICH downregulated mammalian target of rapamycin (mTOR) expression; mTOR is an important mediator of autophagy activation (Yu et al., 2017). Autophagy is also noted in clinical reports. Brain tissue specimens collected from 27 patients with ICH and analyzed by transmission electron microscopy revealed that autophagosomes and autolysosomes exist in neurons surrounding the hematoma and that the extent correlates positively with the severity of brain injury (Wu et al., 2019). It is proposed that autophagy may play different roles at stages of ICH, where excessive autophagy may promote early brain injury, but that autophagy may be neuroprotective in the later stages through clearance of cellular debris (Duan et al., 2017; Figure 5).

### Ferroptosis

Ferroptosis is a non-apoptotic form of regulated cell death (Xie et al., 2016). It was first reported in 2012 and characterized by the iron-dependent accumulation of toxic lipid reactive oxygen species, which was presumed to lead to irreparable lipid damage and membrane permeabilization, ultimately resulting in membrane disorganization and non-specific membrane perforation (Magtanong and Dixon, 2019). It can be elicited by inhibiting the cystine/glutamate antiporter, system Xc^–^ or by loss of activity of glutathione peroxidase 4 (Imai et al., 2017). It is associated morphologically with mitochondrial fragmentation and cristae enlargement (Friedmann et al., 2014; Cao and Dixon, 2016; Gao et al., 2019). Distinguishing itself from other types of cell death, ferroptosis does not depend on energy consumption, caspases, and lysosome activation (Dixon et al., 2012; Stockwell et al., 2017).

**FIGURE 6:**
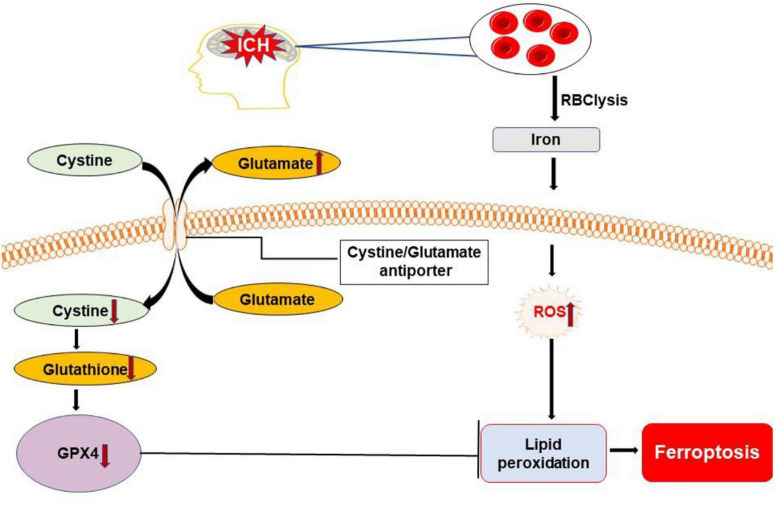
Overview of ferroptosis pathways following ICH. Dysfunction of the cystine/glutamate antiporter after ICH leads to decreased synthesis of glutathione (GSH) and activity of glutathione peroxidase 4 (GPX4). Iron released from lysed erythrocytes can produce highly toxic hydroxyl radicals to attack DNA, proteins, and lipid membranes. The deficiency of GPX4 combined with the presence of toxic iron leads to the accumulation of lipid peroxides and the execution of ferroptosis. ROS, reactive oxygen species.

Iron is a major product of lysed erythrocytes in hematoma. It can form highly toxic hydroxyl radicals to attack DNA, proteins, and lipid membranes, thereby disrupting cellular functions and causing neuronal death. Iron is required for the accumulation of lipid peroxides and the execution of ferroptosis (Stockwell et al., 2017). Very recent evidence suggest that ferroptosis occurs in a mouse model of ICH where it contributes to neuronal death (Figure 6). Li et al. (2017) observed shrunken mitochondria in soma and axons at the margin of the hematoma at both the 3 and 6 days after ICH; swollen mitochondria were observed in the cytoplasm, but not in axons at both the time points. Iron released from hemoglobin triggers reactive oxygen species formation, which also favors the induction of ferroptosis (Magtanong and Dixon, 2019). Moreover, neuronal death displayed morphology and molecular features of ferroptosis, which was corroborated by increased phosphoextracellular signal-regulated kinase levels 6–24 h following hemin treatment (Zille et al., 2017). In addition, recent studies show that ferroptosis is closely associated with inflammation, whereby inhibiting ferroptosis alleviates inflammation after ICH (Zhang et al., 2018; Sun et al., 2020).

### Parthanatos

Poly(ADP-ribose) polymerase-1 (PARP-1)-dependent cell death, known as parthanatos, is unique in terms of its biochemical and morphological features, including rapid PARP-1 activation, early poly(ADP-ribose) (PAR) accumulation, depolarization of mitochondria, early translocation of nuclear apoptosis-inducing factor, loss of cellular nicotinamide adenine dinucleotide (NAD) and ATP, and late caspase activation (Xu et al., 2019).

Microglia/macrophages, thrombin, matrix metalloproteinases, and hematoma degradation products may initiate all the parthanatos. *In vitro*, activated microglia can cause DNA damage, the translocation of apoptosis-inducing factors from mitochondria to the nucleus, mitochondrial membrane permeabilization, and PARP-1 elevation (Porte et al., 2018). Recently, one study reported that PARP-1 may serve an important role in regulating the inflammatory response *via* the nuclear factor-κB pathway in subarachnoid hemorrhage (Satoh et al., 2001; Fan et al., 2019). Another study showed that after hemoglobin infusion to mimic ICH, PARP-1 was activated (Bao et al., 2008).

Oxidative stress is an important pathway to cause DNA damage and brain injury after ICH (Nakamura et al., 2005; Chen K.H. et al., 2019; Shao et al., 2019). It is a critical factor in triggering parthanatos. Moreover, PARP-1 activation is considered as a hallmark of oxidative stress (Robinson et al., 2019). Oxidative stress causes the activation of PARP-1 generating PAR that triggers the release of mitochondrial apoptosis-inducing factors to initiate parthanatos (Zhang et al., 2017).

**FIGURE 7:**
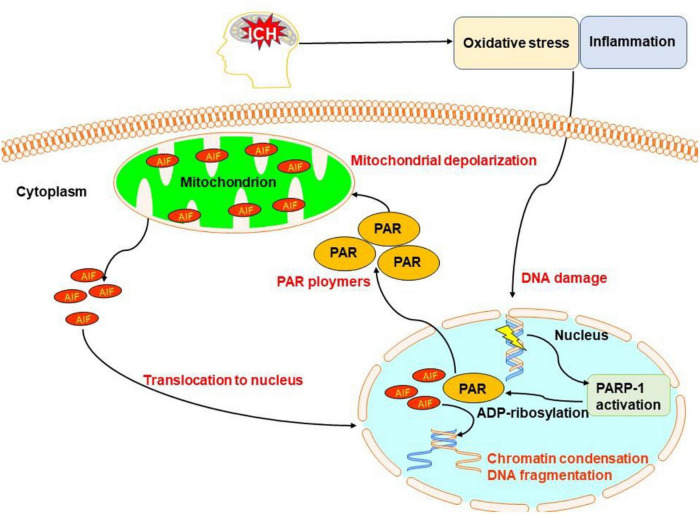
Overview of pathanatos pathways following ICH. Oxidative stress and inflammation after ICH damages DNA, leading to the activation of poly(ADP-ribose) polymerase-1 (PARP-1), which catalyzes the excessive synthesis of PAR intracellularly. PAR directly interacts with the C-terminus of membrane-bound apoptosis-inducing factor and triggers the release of apoptosis-inducing factor (AIF) from the mitochondria. AIF then begins its journey toward the nucleus and activates caspases causing chromatin condensation and DNA fragmentation.

Altogether, parthanatos may be triggered by excessive DNA damage induced by oxidative stress, hypoxia, and inflammatory cues, which are the key pathologic process of ICH (Figure 7). The exact pattern of parthanatos in ICH remains unclear. The time course and region, as well as target cell type of parthanatos, after ICH is still unclear. Much study is required to identify the role of parthanatos in ICH.

## Treatment of ICH With Anticell Death Agents

In this section, we discuss agents targeting individual-specific cell death pathways. We also review multitargets strategies, such as anti-inflammatory and/or antioxidative stress for ICH. This may provide the impetus to translate anticell death strategies in models into clinical applications.

### Targeting Apoptosis in ICH

zVADfmk, a broad-spectrum caspase inhibitor, significantly reduced the density of TUNEL-positive cells (Matsushita et al., 2000) and activate caspase-3 at 24 and 48 h after rat ICH (Kato et al., 2007). Tauroursodeoxycholic acid (TUDCA), an endogenous bile acid, reduced TUNEL-positive stained cells and protected against neurological injury after ICH in rats; the proposed mechanisms are through preserving mitochondrial membrane stability and inhibiting caspase activation (Rodrigues et al., 2003). TUDCA reduced lesion volume and decreased TUNEL-positive stained cells adjacent to hematoma, which was associated with an inhibition of caspase activity of approximately 50%. TUDCA treatment also increased the expression of certain antiapoptotic b-cell leukemia/lymphoma 2 (Bcl-2) family members, inhibited cytochrome c release from mitochondria, decreased nuclear factor-κB activity, and improved neurobehavioral deficits; these features are correspondent with the reduction of apoptotic brain cells (Kluck et al., 1997; Yang et al., 1997). Moreover, TUDCA is also an endoplasmic reticulum stress inhibitor, which could suppress ICH-induced secondary brain injury by regulating autophagy and inhibiting caspase-12-mediated apoptosis after ICH (Duan et al., 2017). TUDCA has also been demonstrated to be a potent strategy to reduce neuronal pyroptosis (Chen et al., 2020).

### Targeting Necrosis in ICH

AdipoRon, an activator of adiponectin receptor 1, attenuates mitochondrial dysfunction after ICH (Kato et al., 2007). It reduced neuronal necrosis as determined by propidium iodide staining, enhanced ATP levels, and reduced reactive oxygen species levels in the perihematomal tissues at 72 h after ICH in mice (Kato et al., 2007). Correspondingly, AdipoRon significantly alleviated OxyHb-induced collapse of mitochondrial transmembrane permeabilization measured by a fluorescence tetraethylbenzimidazolylcarbocyanineiodide (JC-1) kit and it enhanced mitochondrial mass as quantified by a mitochondrial fluorescent probe. Moreover, neurons treated with AdipoRon showed lower necrotic and apoptotic rates as indicated by Annexin V-fluorescein isothiocyanate (FITC)/propidium iodide staining. The authors further demonstrated that AdipoRon alleviated necrosis/apoptosis after ICH through an adiponectin receptor 1-mediated pathway (Yu et al., 2019).

### Targeting Necroptosis in ICH

Necrostatin-1, an antinecroptosis chemical, improved neurological function and attenuated brain injury by specifically inhibiting RIP1 and RIP3 in a mouse model of ICH (Su et al., 2015). It protected against hemoglobin- and hemin-induced toxicity in cultured neurons (Zille et al., 2017). Necrostatin-1 can also suppress apoptotic and autophagic pathways to exert neuroprotection in mice with ICH (Chang et al., 2014). In addition, metformin, a powerful medication for the treatment of type 2 diabetes, attenuated neuronal necroptosis *via* activating AMP-activated protein kinase signaling (Lin et al., 2021). Moreover, melatonin, a hormone that is predominantly synthesized and secreted from the pineal gland, suppressed RIP3-mediated microglial necroptosis by affecting the deubiquitinating A20 enzyme (Lu et al., 2019).

### Targeting Pyroptosis in ICH

Nucleotide-binding oligomerization domain-like receptor pyrin domain-containing 1-dependent neuronal pyroptosis can be decreased by CCR5 antagonist maraviroc and melanocortin receptor 4 agonist RO27-3225; this is reported to be through CCR5/PKA/CREB pathway and apoptosis signalling-regulating kinase 1 (ASK1)/jun N-terminal kinase (JNK)/p38 mitogen-activated protein kinase (MAPK) pathways after ICH in mice, respectively (Chen S. et al., 2019; Yan et al., 2021). Microglia pyroptosis induced by ICH was suppressed by hypoxia-preconditioned olfactory mucosa mesenchymal stem cells or pharmacological treatment with andrographolide, by inhibiting the NLRP3 inflammasome and caspase-1 activity (Li et al., 2018d; Liu J. et al., 2021). AC-YVAD-CMK, a selective inhibitor of caspase-1, reduced caspase-1 activation and inhibited IL-1 production and maturation, but has no effect on NLRP3 expression. Furthermore, the AC-YVAD-CMK-treated mice had a reduction in proinflammatory microglia polarization and improved recovery of neurological function (Lin et al., 2018).

### Targeting Autophagy in ICH

Rapamycin, a strong inducer of autophagy by blocking mTOR, decreased brain injury and neuronal death surrounding the hematoma at 7 days after ICH in rats (Li et al., 2016). However, in another collagenase-induced ICH mouse model, rapamycin increased brain edema and the number of propidium iodide-positive cells *in vitro* and 24–72 h after ICH *in vivo* (Shen et al., 2016). As mentioned above, these contrary results may be due to the differential functions of autophagy at different stages of ICH. Therefore, strategies targeting autophagy after ICH need to be evaluated carefully.

### Targeting Ferroptosis in ICH

Ferrostatin-1, a specific inhibitor of ferroptosis, has been discovered to prevent mitochondria shrinkage, reduce iron deposition, and inhibit prostaglandin-endoperoxide synthase, cyclooxygenase-2 (COX-2), and malondialdehyde levels. Additionally, ferrostatin-1 improved ICH-induced neurological deficits, memory impairment, and brain atrophy (Chen B. et al., 2019). Moreover, ferrostatin-1 in combination with other inhibitors that target different forms of cell death prevented hemoglobin-induced cell death in hippocampal slice cultures and human-induced pluripotent stem cell-derived neurons better than any inhibitor alone (Li et al., 2017).

## Multimodal Anticell Death Strategies for ICH

Hydrogen inhalation has been reported to selectively scavenge hydroxyl radicals and preserve the blood-brain barrier disruption by preventing mast cell activation after ICH (Manaenko et al., 2013). It was also reported to reduce the number of TUNEL-positive cells; inhibit caspase-3; lower levels of TNF, IL-1, and brain-derived neurotrophic factor (BDNF) messenger RNA (mRNA); and decrease malondialdehyde content after experimental ICH in rats (Choi et al., 2018).

Hydrogen sulfide is an important gasotransmitter for NLRP3 inflammasome (Zhao et al., 2017). Recent research found that endogenous hydrogen sulfide production was downregulated after ICH due to decreased cystathionine-β-synthase in the brain. Treatment with sodium hydrosulfide, a hydrogen sulfide donor, attenuated brain edema, injury volume, and neurological deficits by blocking caspase-3 cleavage, elevating Bcl-2, and suppressing the activation of autophagy marker (LC3II and Beclin-1) in the injured striatum post-ICH (Shan et al., 2019). Sodium hydrosulfide also attenuated brain edema, microglial accumulation, and neurological deficits at 1 day post-ICH by inhibiting the P2X7R/NLRP3 inflammasome cascade (Zhao et al., 2017).

Free radicals may contribute to cell death and brain injury in ICH. Treatment with the free radical trapping agent disodium 4-[(tert-butylimino)methyl]benzene-1,3-disulfonate N-oxide (NXY-059) following ICH in rats significantly reduced the neutrophil infiltration and the number of TUNEL-positive cells observed at 48 h adjacent to the hematoma; it significantly decreased neurological impairment on days 1 through 21 (Peeling et al., 2001). This has brought to the clinic where 607 patients with acute ICH were administered NXY-059. The results showed good safety and tolerability for NXY-059, but there were no differences in 3-month function, disability, or neurological deficit scores (Lyden et al., 2007).

Deferoxamine, an iron chelator, may penetrate the blood-brain barrier and accumulate in brain tissues after systemic administration (Hua et al., 2008). Chelated ferric iron and hemosiderin can form a stable complex with deferoxamine, thereby preventing iron from entering the Haber–Weiss reaction for the production of hydroxyl radicals to induce apoptosis (Selim, 2009). In a collagenase-induced ICH model, systemic administration of deferoxamine decreased iron accumulation and neuronal death, attenuated production of reactive oxygen species, and reduced microglial activation and neutrophil infiltration (Selim, 2009). In a clinical trial with 42 patients with ICH, brain edema was significantly inhibited by deferoxamine treatment (Yu et al., 2015). More recent clinical trial data have confirmed the safety of deferoxamine infusion at 32 mg/kg/day for three consecutive days in patients with ICH (Selim et al., 2019). Following a *post hoc* analysis of the intracerebral hemorrhage deferoxamine trial (iDEF Ttrial), a greater proportion of deferoxamine than placebo-treated patients achieved favorable outcomes in patients with moderate hematoma volume (10–30 ml) (Wei C. et al., 2021).

Minocycline, a tetracycline derivative that can cross the blood-brain barrier, significantly reduced neutrophil and macrophage infiltration, microglia activation, cell death, and improved neurobehavioral outcomes after ICH in models (Sheng et al., 2018; Li et al., 2021). Our recent research showed that minocycline decreased Fluoro-Jade C-positive cells, which depict dying neurons (Schmued, 2016) and reduced TUNEL-positive cells, in part by inhibiting the extracellular matrix metalloproteinase inducer/matrix metalloproteinase-9 pathway (Liu Y. et al., 2021). Moreover, the combination of minocycline and deferoxamine improves their individual neuroprotective effect on acute ICH in rats (Li et al., 2021). A study in the clinical trial of minocycline in ICH showed that 400 mg dose of minocycline was safe and achieved neuroprotective serum concentrations (Fouda et al., 2017).

## Conclusion and Future Directions

Intracerebral hemorrhage is a leading medical problem without any effective treatment options. An imbalanced oxidative stress response and overactivated inflammatory cascade trigger several cell death pathways in and around the brain hematoma following ICH. These modes of cell death comprise apoptosis, necrosis, necroptosis, pyroptosis, ferroptosis, autophagy, parthanatos, and likely others to be discovered. Strategies targeting these cell death pathways have resulted in neuroprotection in preclinical models and some of these have shown promise for patients with ICH.

There remain many challenges and future directions in modulating cell death to improve the prognosis of patients with ICH. First, as far as we know, brain cell death could not be documented non-invasively *in vivo*; such capacity would be important for the extension of anticell death strategies from bench to clinic. Exquisite non-conventional MRI or PET may offer potential methods to evaluate the mode of brain cell death in preclinical or clinical ICH. In addition, many of the medications presented here are tested in culture or for short periods in an animal model and they focus on limited molecular targets. Longer-term and comprehensive preclinical studies and, perhaps, modulating several modes of cell death would be needed before a candidate drug is translated into clinical trials. Moreover, whether a particular cell death pathway is more dominant than others is worthy of investigation, as a predominant hierarchical target could lead to more favorable outcomes.

Notably, ICH is a complex disorder involving several types of cell death pathways evolving in different stages or regions and the cell death pathway is closely associated with multiple reciprocal pathophysiological or compensatory pathways. Nonetheless, with continuing research, it is worth contending that anticell death strategies, especially the multitargets anticell death treatments that also have anti-inflammatory and antioxidative activity, may become available as powerful protectants against ICH-induced brain cell death.

## Search Strategy and Selection Criteria

Document retrieval was executed through PubMed and Clinical Trials by the National Institutes of Health. The keywords “cell death,” “apoptosis,” “necrosis,” “necroptosis,” “pyroptosis,” “ferroptosis,” “autophagy,” “parthanatos,” and “intracerebral hemorrhage” or “ICH” were employed to identify all the full-text articles in English. Cited references were searched and retrieved for potentially eligible publications containing cell death of ICH. All the subarachnoid hemorrhage, traumatic brain hemorrhage, cancerous cerebral hemorrhage, and studies, which included inappropriate cell death detection methods, were excluded.

## Author Contribtions

All the authors wrote and revised the final version of the manuscript.

## Conflict of Interest

The authors declare that the research was conducted in the absence of any commercial or financial relationships that could be construed as a potential conflict of interest.

## Publisher’s Note

All claims expressed in this article are solely those of the authors and do not necessarily represent those of their affiliated organizations, or those of the publisher, the editors and the reviewers. Any product that may be evaluated in this article, or claim that may be made by its manufacturer, is not guaranteed or endorsed by the publisher.
